# Supplementary figures and images for: Exploring causal effects of smoking and alcohol related lifestyle factors on self-report tiredness: A Mendelian randomization study
Source: PLoS One. 2023 Jun 16;18(6):e0287027. doi: 10.1371/journal.pone.0287027 (PMC10275431; doi:10.1371/journal.pone.0287027)

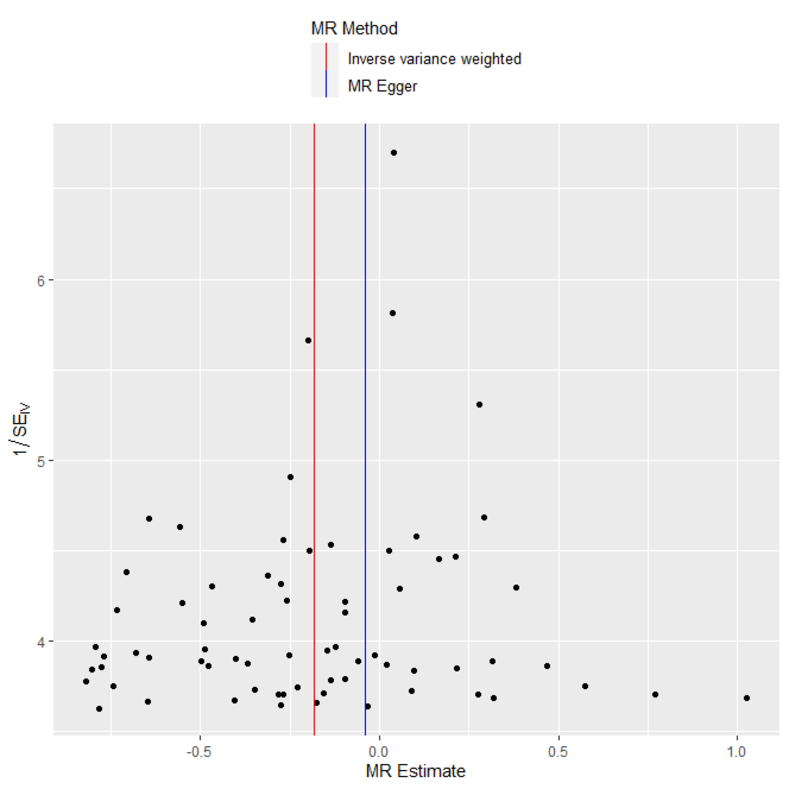

Supplement: S1 File — Fig A. Funnel plots for the effect of never smoking status on risk of self-reported fatigue for each single-nucleotide polymorphism (SNP), the resulting Mendelian randomization (MR) estimate is plotted against the inverse of the standard error of the MR estimate. Symmetry noted in this plot provides evidence against the presence of directional horizontal pleiotropy. The inverse-variance weighted and MR Egger causal estimates are represented by a red and blue line respectively. Fig B. Funnel plots for the effect of current smoking status on risk of self-reported fatigue for each single-nucleotide polymorphism (SNP), the resulting Mendelian randomization (MR) estimate is plotted against the inverse of the standard error of the MR estimate. Symmetry noted in this plot provides evidence against the presence of directional horizontal pleiotropy. The inverse-variance weighted and MR Egger causal estimates are represented by a red and blue line respectively. Fig C. Funnel plots for the effect of alcohol intake frequency on risk of self-reported fatigue for each single-nucleotide polymorphism (SNP), the resulting Mendelian randomization (MR) estimate is plotted against the inverse of the standard error of the MR estimate. Symmetry noted in this plot provides evidence against the presence of directional horizontal pleiotropy. The inverse-variance weighted and MR Egger causal estimates are represented by a red and blue line respectively. (ZIP) [file pone.0287027.s002.zip › Fig A.tif]

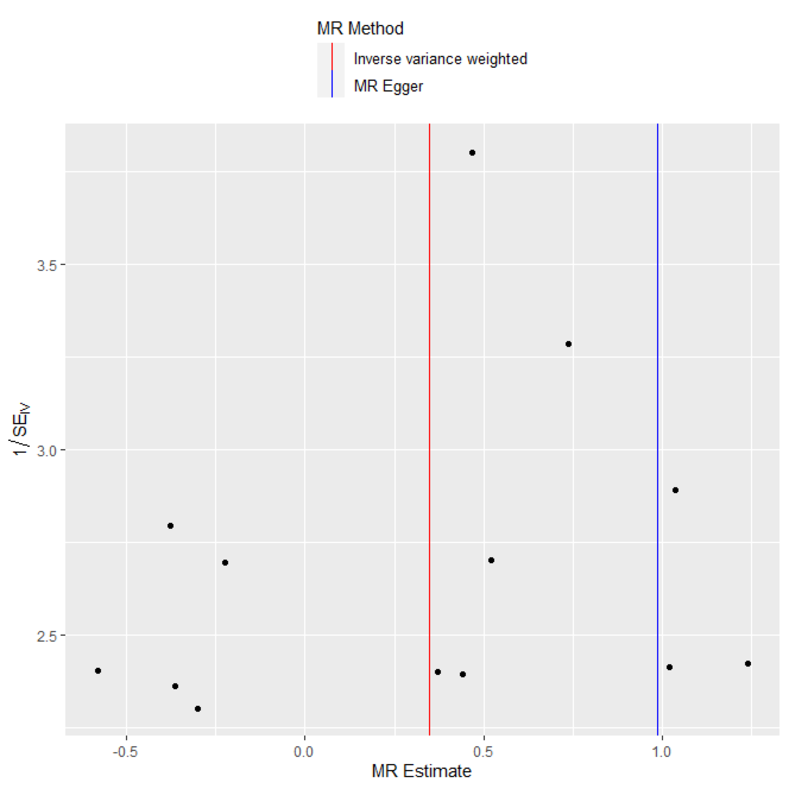

Supplement: S1 File — Fig A. Funnel plots for the effect of never smoking status on risk of self-reported fatigue for each single-nucleotide polymorphism (SNP), the resulting Mendelian randomization (MR) estimate is plotted against the inverse of the standard error of the MR estimate. Symmetry noted in this plot provides evidence against the presence of directional horizontal pleiotropy. The inverse-variance weighted and MR Egger causal estimates are represented by a red and blue line respectively. Fig B. Funnel plots for the effect of current smoking status on risk of self-reported fatigue for each single-nucleotide polymorphism (SNP), the resulting Mendelian randomization (MR) estimate is plotted against the inverse of the standard error of the MR estimate. Symmetry noted in this plot provides evidence against the presence of directional horizontal pleiotropy. The inverse-variance weighted and MR Egger causal estimates are represented by a red and blue line respectively. Fig C. Funnel plots for the effect of alcohol intake frequency on risk of self-reported fatigue for each single-nucleotide polymorphism (SNP), the resulting Mendelian randomization (MR) estimate is plotted against the inverse of the standard error of the MR estimate. Symmetry noted in this plot provides evidence against the presence of directional horizontal pleiotropy. The inverse-variance weighted and MR Egger causal estimates are represented by a red and blue line respectively. (ZIP) [file pone.0287027.s002.zip › Fig B.tif]

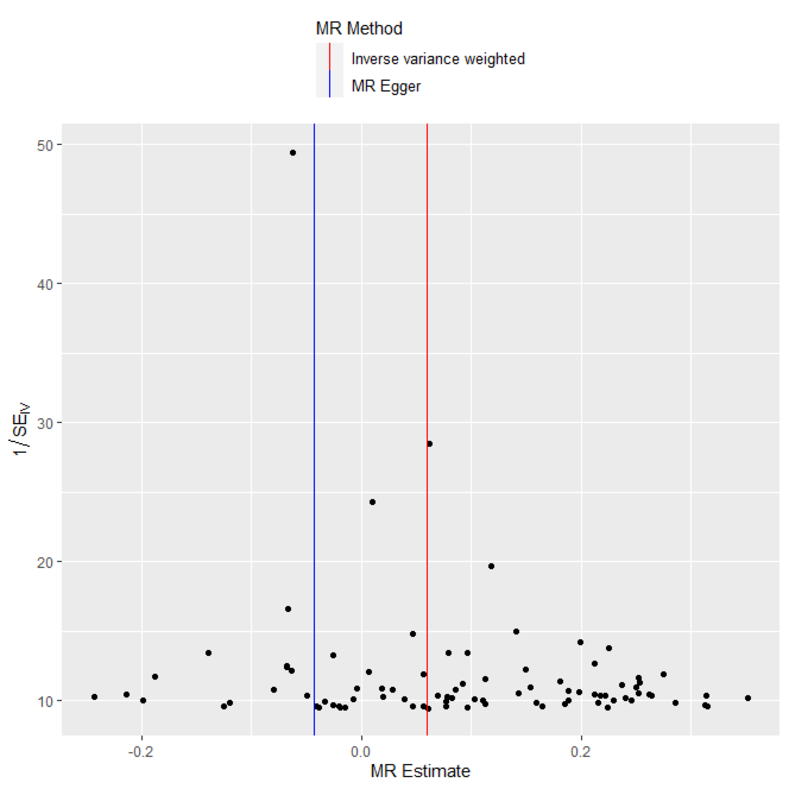

Supplement: S1 File — Fig A. Funnel plots for the effect of never smoking status on risk of self-reported fatigue for each single-nucleotide polymorphism (SNP), the resulting Mendelian randomization (MR) estimate is plotted against the inverse of the standard error of the MR estimate. Symmetry noted in this plot provides evidence against the presence of directional horizontal pleiotropy. The inverse-variance weighted and MR Egger causal estimates are represented by a red and blue line respectively. Fig B. Funnel plots for the effect of current smoking status on risk of self-reported fatigue for each single-nucleotide polymorphism (SNP), the resulting Mendelian randomization (MR) estimate is plotted against the inverse of the standard error of the MR estimate. Symmetry noted in this plot provides evidence against the presence of directional horizontal pleiotropy. The inverse-variance weighted and MR Egger causal estimates are represented by a red and blue line respectively. Fig C. Funnel plots for the effect of alcohol intake frequency on risk of self-reported fatigue for each single-nucleotide polymorphism (SNP), the resulting Mendelian randomization (MR) estimate is plotted against the inverse of the standard error of the MR estimate. Symmetry noted in this plot provides evidence against the presence of directional horizontal pleiotropy. The inverse-variance weighted and MR Egger causal estimates are represented by a red and blue line respectively. (ZIP) [file pone.0287027.s002.zip › Fig C.tif]
