# Supplementary material for: Exploring causal effects of smoking and alcohol related lifestyle factors on self-report tiredness: A Mendelian randomization study
Source: PLoS One. 2023 Jun 16;18(6):e0287027. doi: 10.1371/journal.pone.0287027 (PMC10275431; doi:10.1371/journal.pone.0287027)
Supplement: Attachment — Submitted filename: response to reviewer.docx [file pone.0287027.s003.docx]

Response to reviewer

Reviewer #1:

1. Plos One is an interdisciplinary journal and thus not all readers might be familiar with the MR method. Therefore, the idea of the two-sample MR and MR, in general, should be explained.

Please refer to the corresponding part of revised manuscript (method section on pages 3 and 4.).

2. In the two-sample MR, the evidence on the associations of genetic variants with the risk factor and the outcome should come from nonoverlapping data sources. It seems that in this study, both associations come from the same sample (UKBB). Don’t the samples overlap? If so, what are the consequences?

Please refer to the corresponding part of revised manuscript (Strengths and limitations section on page 15).

3. (a) There are very few citations to the previous literature regarding the relationship between smoking, alcohol intake and fatigue. For example, when discussing the relationship between alcohol intake and fatigue, the authors only refer to Woolley et al. (2004) and Vella et al. (2010).

(b) Furthermore, these articles seem to be off the topic. Woolley et al. examine whether chronic fatigue syndrome patients reduce or cease alcohol intake, and Vella et al. examine how alcohol consumption is related to exercise performance and recovery. In this paper, the authors examine whether alcohol consumption causes fatigue.

(c) It was also unclear how the study by Juster et al. (2010) was related to the authors' topic regarding smoking and fatigue.

In the following part, I will give you the explanation corresponding to the comments above step by step.

(a) As you mentioned, there are very few citations to the previous literature. There are indeed very few literatures on the topic. We have investigated the subject via searching on the PubMed and Google scholar websites, and indeed found very few literatures on such topic.

(b) I found the topic of Woolley et al. is related to mine, if you don’t mind me saying so. In the abstract of their article, Woolley et al. gave the reason for reducing alcohol intake [The most common reasons were ***increased tiredness after drinking (67%)***, increased nausea (33%), exacerbated hangovers (23%) and sleep disturbance (24%)]. Their findings support my research on the basis of the association between fatigue and alcohol intake.

Vella et al. reviewed how alcohol consumption is related to exercise performance (I). The exercise performance includes endurance performance (II), which is a core features to define physical fatigue (III), i.e. the inability to continue an endurance activity. My study is to investigate whether there is the causal relationship between alcohol consumption and (III) which is directly related to (II) and partly related to (I).

(c) I explained how the study by Juster et al. was related to my topic on page 12 based on the content of Juster et al. (2010). ***In section 4.2***, Juster et al. gave us the following ***two findings: The first one is that smoking interacts with greater allostatic load. Another one is that increased fatigue is related to higher allostatic load.***

4. There is evidence that alcohol consumption is related to poorer sleep. Could that also explain the positive link between alcohol consumption and fatigue?

No. The reason is that alcohol consumption may have negative horizontal pleiotropy related to fatigue via other mediators rather than sleep.

5. In Figure 4, the authors report the Wald ratio for the “never drinking” outcome. In the table notes, the authors should inform the reader why this statistic was reported in this case.

See Figure 4 note in revised manuscript on page 11.

6. How many SNPs were related to self-reported tiredness? This was not mentioned in the section "Data sources and processing".

See “Data sources and processing” section in revised manuscript on page 6.

7. Some of the abbreviations were not explained in the text.

We examined the abbreviations in the manuscript carefully and revised.

Reviewer #2:

1. I am very appreciated for your valuable suggestions. Multivariable Mendelian Randomization was performed as additional sensitivity analyses to test the robustness of the findings from univariate MR in method section on page 8. Other sections were revised correspondingly on pages 6, 10, 11.
2. I am very sorry to tell you I have no other clues to check the direction for the alcohol intake frequency data in the UK Biobank. But the finding, i.e. the causal effect of alcohol intake frequency and alcohol drinking states on fatigue, might provide some evidence to support your guess – higher categories correspond to reduced alcohol intake frequency.
