# Supplementary material for: Exploring causal effects of smoking and alcohol related lifestyle factors on self-report tiredness: A Mendelian randomization study
Source: PLoS One. 2023 Jun 16;18(6):e0287027. doi: 10.1371/journal.pone.0287027 (PMC10275431; doi:10.1371/journal.pone.0287027)
Supplement: Attachment — Submitted filename: response to reviewer.docx [file pone.0287027.s004.docx]

Response to reviewer

Reviewer #1:

I am very appreciated for your serious and careful review as this is my first paper in bioinformatics. With your help, I will achieve a satisfied paper, and Mendelian Randomization will become a flexible tool for me. I believe I have made much progress. Thank you again for your time!

1. Out of the blue, at the beginning of the Data Sources and Processing section (page 5, line 100), the authors start discussing psychiatric disorders. The rationale for using these variables should have been given much earlier. What is the purpose of using these variables?

Please refer to the corresponding part of revised manuscript (introduction section on page 3, line 68-71.).

2. The interpretation of the Multivariable Mendelian Randomization (MVMR) results needs clarification.
（a） Could you please explain why MVMR was performed?

Please refer to the corresponding part of revised manuscript (introduction section on page 3, line 68-71.). I explained the reason for performed MVMR.

（b） Was it to investigate whether the link between SAIEs and fatigue was mediated by psychiatric disorders?

No, this paper is to investigate whether there is a direct link between SAIEs and fatigue not via psychiatric disorders. In fact, I intend to exclude the effect of mental illness on the direct casual estimate between SAIEs and fatigue. I apply the MVMR method which excludes psychiatric disorders as confounders and mediators.

（c） Are the reported estimates the remaining direct effects after controlling for the mediated pathway via psychiatric disorders?

Yes, as you mentioned, MVMR results show the remaining direct effects after controlling for the mediated pathway via psychiatric disorders. Besides this, the method MVMR also eliminated the possibility of psychiatric disorders to be confounders for SAIEs and fatigue.

（d） If so, why do the authors indicate on page 10 (lines 211-212) that the MVMR results did not support the idea of a causal relationship between current smoking and fatigue?

For the exposure (current smoking: status), the confidence interval for the odds ratio obtained from MVMR is including “1”, i.e., “1” represents no direct causal relationship; on the contrary, the same parameter from univariate MR is not including “1”.

（e） It should be noted that a mediated effect via psychiatric disorders is still part of the causal effect. Even if there is no direct effect after controlling for a mediated effect, a significant total effect may still exist. It seems to me that MVMR was performed to provide an explanation for the relationship between SAIEs and fatigue.

As you mentioned, ***if there is no overlapping between the instrument variables (SNPs) for exposures***, MVMR findings can provide an explanation for the relationship between SAIEs and fatigue. But the instrument variables of SAIEs are quite strong and contain genetic variants ***located throughout the genome***, they may have corresponding associations with psychiatric endpoints. Therefore, generally there is overlapping SNPs between SAIEs and psychiatric disorders. Such overlapping SNPs lead to false positives causal estimate of univariate MR.

3. Regarding the previous comments, could you please clarify the meaning of the following sentence (page 10, lines 212-215): The discrepancy with our results – suggesting mediation or confounding by psychiatric disorders – highlights the importance of the inclusion for all the SNPs significantly associated with substance use and psychiatric disorders in multivariable MR analysis, to prevent loss of precision and potentially even biased estimates.”

The discrepancy with our results, i.e., there are different results between MVMR and univariate MR for the exposure (current smoking: status).

suggesting mediation or confounding by psychiatric disorders gives the reason for the discrepancy with our results (MR and MVMR). More clearly the discrepancy is due to the two basic relationships (confounder and meditator) between psychiatric disorders and SAIEs.

the inclusion for all the SNPs significantly associated with substance use and psychiatric disorders in multivariable MR analysis, i.e., one assumption of MVMR (exposures must be strongly predicted by the SNPs “given the other exposures included in the model”). This assumption ensures MVMR to eliminate possible bias from mediation or confounding by psychiatric disorders in univariate MR.

4. The MVMR results were added to the methods and results sections without being fully integrated into the rest of the paper. The authors should revise the paper to better incorporate the MVMR results and their implications into the overall discussion and conclusions.

I added corresponding revision in the discussion section on page 12 (line 257-258, 261-262,265-271), page 13 (291-292), page 14 (line 293-294, line 307-308), page 15 (line 329); and in conclusion section on page 16, line 338.

5. Page 7, line 140: “to avoid the violation of the assumptions 2 and 3, we assessed instrument strength…” Instrument strength is related to assumption 1. Could you please clarify why assumptions 2 and 3 are mentioned here?

The effect of instrument strength on MR test is not only related to assumption 1. Weak instruments that poorly predict the risk factor can amplify the bias due to violations of the core instrumental variable assumptions, i.e., horizontally pleiotropic effects of variants and confounders.

6. On page 7, line 144, the statement 'To test whether all the IVs satisfy the IV assumptions…' needs clarification. Could you please specify which assumptions you are referring to?"

Please refer to the revised corresponding part: Page 7, line 148.

7. Fig 1: It appears that IV assumption 2 and 3 should be the other way around.
   Sorry, I am not sure if I understand this comment fully. In fact, there is no fixed order for assumption 2 and 3. I checked my paper carefully and searched figures relevant to MR through internet. Most figures about assumption 2 and 3 are in the same order of mine.
8. Page 6 line 126: should the p-value be p < 0.05 instead of p > 0.05?

Yes, the p-value should be p < 0.05. Thanks a lot!

9. Pages 8-9 lines 181-182. The authors referred to Corwin et al. (2002) as a reference for the multivariable MR method assumptions, but this paper is not relevant to the multivariable MR method. Could you please provide a better reference for the multivariable MR method assumptions?

I am very sorry for this typesetting error. In fact, the order of Corwin et al. (2002) in the reference list is 31 in the paper revised last time. Another reference no.30 is the corresponding one for MVMR method in that paper.

10. The authors reported the results for never-smoking status first, followed by current smoking status in the text, but the order of the results is reversed in Figure 2 (and a similar situation in Figure 3). To maintain consistency, the results should be presented in the same order in both the text and tables.

Please to refer the revised Figure 2 and 3.

11. Page 10 line 212: I believe that “multivariable IV MR” should be “IVW multivariable MR analysis” for consistency.

Thanks for your help! Please refer to the revision on page 10 (line 216).

12. Figure 4: There appears to be a discrepancy between the information in the legend and the footnote. According to the legend, the orange results indicate the MVMR results, but the footnote indicates that the blue results report the results adjusted for psychiatric disorders.

Thanks for your help, please refer to the revision on page 11 (line 232).

13. The explanation for the abbreviation PD is missing in the notes of Fig 5.

Thanks for your help! Please refer to the revision on page 11 (line 235).

14. The phrase "we found strong evidence" should be toned down as the MR method has its limitations, which may not have been fully addressed in this study.

Please refer to the revision on page 12 (line 257). Thank you !

15. In the limitations section, the authors should acknowledge that the presence of pleiotropy as a potential factor affecting their results cannot be ruled out.

please refer to the revision on page 15 (line 329). Thank you!
